# Computational Modeling and Experimental Investigation of CO_2_-Hydrocarbon System Within Cross-Scale Porous Media

**DOI:** 10.3390/molecules30020277

**Published:** 2025-01-12

**Authors:** Feiyu Chen, Linghui Sun, Bowen Li, Xiuxiu Pan, Boyu Jiang, Xu Huo, Zhirong Zhang, Chun Feng

**Affiliations:** 1 University of Chinese Academy of Sciences, Beijing 100049, China; chenfeiyu23@mails.ucas.ac.cn (F.C.); libowen201@mails.ucas.ac.cn (B.L.); panxiuxiu22@mails.ucas.ac.cn (X.P.); jiangboyu24@mails.ucas.ac.cn (B.J.); huoxu21@mails.ucas.ac.cn (X.H.); zhangzhirong20@mails.ucas.ac.cn (Z.Z.); 2 Institute of Porous Flow and Fluid Mechanics, Chinese Academy of Sciences, Langfang 065007, China; 3 Research Institute of Petroleum Exploration & Development, Beijing 100083, China; fengchun123@petrochina.com.cn; 4 State Key Laboratory of Enhanced Oil & Gas Recovery, Beijing 100083, China

**Keywords:** CO_2_ flooding, multi-scale, phase behavior, enhanced oil recovery, confined fluids

## Abstract

CO_2_ flooding plays a crucial role in enhancing oil recovery and achieving carbon reduction targets, particularly in unconventional reservoirs with complex pore structures. The phase behavior of CO_2_ and hydrocarbons at different scales significantly affects oil recovery efficiency, yet its underlying mechanisms remain insufficiently understood. This study improves existing thermodynamic models by introducing Helmholtz free energy as a convergence criterion and incorporating adsorption effects in micro- and nano-scale pores. This study refines existing thermodynamic models by incorporating Helmholtz free energy as a convergence criterion, offering a more accurate representation of confined phase behavior. Unlike conventional Gibbs free energy-based models, this approach effectively accounts for confinement-induced deviations in phase equilibrium, ensuring improved predictive accuracy for nanoscale reservoirs. Additionally, adsorption effects in micro- and nano-scale pores are explicitly integrated to enhance model reliability. A multi-scale thermodynamic model for CO_2_-hydrocarbon systems is developed and validated through physical simulations. Key findings indicate that as the scale decreases from bulk to 10 nm, the bubble point pressure shows a deviation of 5% to 23%, while the density of confined fluids increases by approximately 2%. The results also reveal that smaller pores restrict gas expansion, leading to an enhanced CO_2_ solubility effect and stronger phase mixing behavior. Through phase diagram analysis, density expansion, multi-stage contact, and differential separation simulations, we further clarify how confinement influences CO_2_ injection efficiency. These findings provide new insights into phase behavior changes in confined porous media, improving the accuracy of CO_2_ flooding predictions. The proposed model offers a more precise framework for evaluating phase transitions in unconventional reservoirs, aiding in the optimization of CO_2_-based enhanced oil recovery strategies.

## 1. Introduction

Since the exploration and production of oil and gas, numerous industries have benefited from their development. However, the increasing CO_2_ emissions associated with the combustion of fossil fuels have exacerbated environmental degradation. In this context, CCS (Carbon Capture and Storage) technology has gradually emerged. However, the high cost of CCS projects under current technological conditions is difficult to bear [1,2]. Therefore, the CCUS (Carbon Capture, Utilization, and Storage) technology, which is based on CO_2_ reuse to reduce costs, is gradually gaining attention. As an important branch of tertiary oil recovery, the relatively low critical temperature and pressure of CO_2_ make it a good displacing medium with good miscibility with crude oil. The injected CO_2_ interacts with the crude oil, resulting in mass transfer diffusion, dispersion, and dissolution phenomena [3]. This process enhances sweeping efficiency and mobilizes crude oil more effectively [4,5]. In addition, CO_2_ can effectively occupy the pore space in the reservoir and remain in the formation during oil and gas field development, contributing to low-carbon and decarbonization goals. Although CO_2_ flooding has achieved certain results in improving the recovery rate in low-permeability and unconventional reservoirs, and has also played a role in CO_2_ sequestration to some extent, the micro-pore structure of reservoirs suitable for CCUS-EOR is complex, with a wide range of pore throat sizes and developed fractures/micro-fractures. The constraints caused by the reduction of pore sizes in porous media may lead to significant changes in physical properties and phase behavior, such as critical pressure and density, viscosity, surface tension, bubble point, dew point, interfacial properties at the fluid-wall interface, and oil–gas interfacial tension [5,6]. This can result in deviations in reservoir scheme design, affecting the actual oil and gas recovery rate and CO_2_ sequestration rate. For example, the bubble point pressure of the confined fluid is lower than that of the bulk oil, thus delaying the process of light hydrocarbon escaping from the oil phase to the gas phase during production. This can explain the positive discrepancy between actual production data from reservoirs rich in nanoscale storage spaces and conventional simulation results in terms of oil production and oil recovery ratio. Currently, there have been numerous studies on the macroscopic property changes and measures such as adjusting the injection profile through Water Alternating Gas (WAG), which provide a certain research basis [7,8,9]. However, the transformation of phase behavior due to the multi-scale effect still has a significant impact on CO_2_ displacement efficiency [10,11]. Recent studies have shown that CO_2_ diffusion in methane-dissolved pore fluids alters phase behavior at multiple scales, influencing CO_2_ transport and utilization in geological storage and enhanced oil recovery (EOR) processes. These effects need to be systematically analyzed to optimize CO_2_ injection strategies. Therefore, clarifying the changes in phase behavior of the CO_2_-reservoir oil system at multiple scales is of great engineering significance for enhancing oil recovery with CO_2_. Various methods have been employed to study the above-mentioned phenomena, including molecular simulations, thermodynamic models, and physical simulations. However, there is still significant room for improvement in existing methods (as shown in Table 1).

The phase behavior of confined fluids depends on the fluid composition in each phase and is evaluated through phase equilibrium calculations. The phase equilibrium calculation process includes phase stability analysis and flash calculations, both of which depend on the fugacity values of each component. In existing models, the calculation of fugacity is based on the equation of state. Therefore, modifications need to be made to the form or usage of the equation of state, such as adding capillary forces to the fugacity equation or modifying the critical parameters of the equation of state, in order to apply the current calculation framework to nano-reservoirs. Currently, there is no commercial simulator that can predict the phase behavior of confined hydrocarbons in porous media with sufficient accuracy and efficiency. Although many researchers have attempted to conduct large-scale simulations using critical displacement, capillary pressure theory, adsorption models, or their combinations [12,13,14], it is still difficult to explain the nature of the driving forces that lead to different phase behaviors. Recent studies utilizing three-dimensional core reconstruction and numerical simulation of CO_2_ displacement in tight oil reservoirs have significantly improved our understanding of phase behavior and transport mechanisms at the reservoir scale. Additionally, microscale stress sensitivity in CO_2_ foam fracturing has been found to influence fluid distribution and displacement efficiency, further impacting the overall phase behavior of CO_2_-oil systems in tight formations [15].

So far, the theoretical calculation methods for the phase behavior of confined fluids at the nanoscale are mostly based on the calculation of Gibbs free energy. However, under the influence of the high capillary pressure caused by the restriction effect, the assumptions of Gibbs free energy under constant temperature and pressure are no longer applicable, which affects the calculation results. Moreover, Gibbs free energy-based methods often require empirical corrections to account for confinement effects, limiting their applicability to nano-reservoirs. To address these limitations, the Helmholtz free energy framework provides a more fundamental thermodynamic approach that inherently incorporates adsorption effects, molecular interactions, and capillary constraints in confined systems. By directly incorporating these effects into the thermodynamic potential, Helmholtz free energy-based models improve the accuracy and stability of phase equilibrium calculations for CO₂-hydrocarbon systems. This approach eliminates the need for empirical corrections and ensures a more robust theoretical foundation for modeling confined phase behavior. Additionally, the current numerical simulation methods that consider scale effects mostly involve constructing fluid numerical simulation algorithms and do not consider the changes in the flow characteristics of confined fluids. In this study, by introducing the Helmholtz free energy as a convergence criterion, improving the existing thermodynamic model, and considering the influence of adsorption on fluid behavior in micro- and nano-scale pores, a multi-scale thermodynamic calculation model suitable for CO_2_-hydrocarbon systems is developed to investigate the influence of scale changes on the phase behavior of CO_2_-hydrocarbon systems. By performing calculations on different block oil data and pressures, the changes in phase behavior parameters during processes such as gas expansion and successive degassing at different scales are simulated, providing a basis for describing the phase behavior of CO_2_-hydrocarbon systems at multiple scale.

**Table 1:** Research methods of phase behavior in confined space.

Method	Subtype	Limitations	Findings	References
Molecular simulation	Molecular Dynamics Simulation (MD)	The study of fluid behavior at the rock core scale remains computationally expensive due to the nanosecond-level time step and the presence of “Lyapunov instability.” Additionally, the effect of pore-throat geometry on fluid behavior is not fully captured	Methane exhibits different diffusion behaviors in nanopores with various geometric structures, with throat size determining its self-diffusion ability	[16]
			The number of adsorbed layers of methane in organic shale nanopores depends on pore size and temperature, where increased temperature weakens methane adsorption	[17]
			In rough nanopores of shale matrix, methane diffusion primarily occurs as planar diffusion, with significant sensitivity to temperature and pressure for rarefied gases	[18]
			The phase behavior and composition distribution of hydrocarbon binary mixtures in heterogeneous nanopores are strongly influenced by nanopore confinement, particularly in smaller pores	[19]
	Monte Carlo (MC)	Monte Carlo methods face challenges in accurately capturing complex molecular interactions, especially near the critical region. Grand Canonical Monte Carlo (GCMC) simulations struggle with phase transition predictions and require correction methods for critical point estimations. Additionally, vapor–liquid equilibrium simulations in nanopores demand extensive computational resources and careful validation against experimental data	Adsorption of methane and ethane in organic shale nanopores is highly dependent on pore size, temperature, and pressure, with a preference for adsorption in smaller pores	[20]
			Competitive adsorption between methane and ethane is observed, with methane showing higher adsorption capacity due to its molecular size and interaction potential	[21]
			Confinement effects in shale reservoirs result in reduced bubble point pressures and increased dew point pressures compared to bulk conditions	[22]
Thermodynamic model	Density Functional Theory (DFT)	DFT simulations often rely on simplified pore structures and assumptions about fluid-wall interactions, which may not fully capture real shale reservoir conditions	Confined fluids exhibit non-uniform density distributions, with higher densities near pore walls	[23]
			The phase behavior of hydrocarbons in nanopores deviates significantly from bulk conditions, with critical pressure and temperature shifting downward	[24]
			Competitive adsorption of hydrocarbons and CO₂ in calcite nanopores influences miscibility pressure and phase equilibrium	[25]
	Thermodynamic Model Modification	The critical displacement equation correlates with the apparent deviations in nanopores but fails to consider the fluid-wall surface interactions separately. Mesoscopic corrections to macroscopic theories are needed to account for wetting effects	The Helmholtz free energy of confined fluids is calculated using a van der Waals mean-field model. Phase behavior shifts depend significantly on pore geometry and wetting properties	[26]
State equation modification	Modified Equation of State	Existing models lack accuracy and efficiency in predicting confined hydrocarbons’ phase behavior. They fail to fully account for adsorption effects, capillary pressure, and fluid–wall interactions, limiting their applicability to shale reservoirs	Adsorption alters the phase equilibrium of confined hydrocarbons, shifting critical temperature and pressure	[27]
			The equation of state is modified to include adsorption effects and capillary pressure in nanopores, improving phase behavior predictions	[28]
			A pressure correction parameter is defined from a microscopic perspective and correlated with the fluid–fluid potential well depth parameter	[29]
			A new mixing rule is proposed to extend the configurational energy to mixtures	[30]
Physical simulation	Adsorption–Desorption Method	Traditional methods often overlook the effect of water saturation on shale pore systems and assume uniform adsorption across all pores	Adsorption behavior varies between organic and inorganic pores. Image recognition and simulation improve understanding of shale gas adsorption–desorption mechanisms. Hysteresis effects are observed, revealing new insights into adsorption dynamics	[31,32,33]
	Differential Scanning Calorimetry (DSC)	Measurements are constrained by the complexity of multicomponent systems, potential errors in heat flow calibration, and the influence of pore size, geometry, and chemical composition	Phase transitions of confined fluids are significantly influenced by nanopore confinement, including shifts in bubble and dew points, and altered thermal behaviors	[34,35,36,37]
	Nanofluidic Control Method	Due to limitations in the observational conditions, a significant amount of experimental work is still required for comprehensive descriptions	The deviation of the saturation point increases as the depth of the nanochannel decreases	[38,39,40]

## 2. Model Establishment

In this section, a thermodynamic model based on the cubic three-parameter equation of state (PR) is established using the Helmholtz free energy, and relevant fluid phase equilibrium calculations are conducted. This includes solving nonlinear equations and stability analysis based on the Helmholtz free energy, in order to achieve fluid equilibrium calculations at a given temperature and pressure. The specific content includes flash calculation equations, mass conservation boundary conditions, and state equations.

The Helmholtz free energy formulation allows direct incorporation of capillary pressure, adsorption effects, and molecular interactions, which are critical for phase equilibrium calculations in confined porous media. Unlike conventional approaches that introduce capillary pressure corrections as external parameters, this method integrates these effects inherently into the thermodynamic framework. As a result, the phase equilibrium calculations are more robust and consistent across different scales. Additionally, this framework ensures numerical stability by formulating equilibrium conditions based on Helmholtz free energy minimization, avoiding convergence issues associated with traditional fugacity-based models in nano-scale systems.

According to thermodynamic specifications, the Helmholtz free energy of the system is in equilibrium between the gas and liquid phases at a specified temperature T, volume V, and mole numbers of Nc components Ni (i = 1, ……, Nc) in the system.

(1) $$dA=dA_{V}+dA_{L}+dA_{VL}=0$$

In Equation (1), *A*, *A_V_*, *A_L_*, and *A_VL_* are the Helmholtz free energies of the entire system, gas phase, liquid phase, and gas–liquid transition zone, respectively.

The different parts of Equation (1) can be expanded based on the variation of the Helmholtz free energy:

(2) $$dA_{V}=-S_{V}dT_{V}-P_{V}dV_{V}+\sum_{i=1}^{Nc}\bar{G_{iV}}dN_{iV}$$

(3) $$dA_{L}=-S_{L}dT_{L}-P_{L}dV_{L}+\sum_{i=1}^{Nc}\bar{G_{iL}}dN_{iL}$$

(4) $$dA_{VL}=-S_{VL}dT_{VL}-P_{VL}dV_{VL}+\sigma da+\sum_{i=1}^{Nc}\bar{G_{iVL}}dN_{iVL}$$

In Equation (2), *S* is entropy, *P* is pressure, *σ* is interfacial tension, *a* is interfacial area, and $\bar{G_{i}}$ is the partial molar Gibbs free energy of component i.

When the Helmholtz free energy of the system is minimized, material conservation should be satisfied:

(5) $$dN_{Li}+dN_{Vi}+dN_{VLi}=0,i=1,\cdot \cdot \cdot ,N_{c}$$

In conclusion, the expression for dA can be obtained:

(6) $$\begin{array}{l} dA=-(P_{V}-P_{L})dV_{V}-(P_{VL}-P_{L})dV_{VL}+\sigma da\\ +\sum_{i=1}^{N_{C}}({\bar{G}}_{iV}-{\bar{G}}_{iL})dN_{iV}+\sum_{i=1}^{N_{C}}({\bar{G}}_{iVL}-{\bar{G}}_{iL})dN_{iVL} \end{array}$$

Since the volume of the gas–liquid transition zone is very small, the variation of components can be neglected, and the above equation can be simplified as

(7) $$dA=-(P_{V}-P_{L})dV_{V}+\sigma da+\sum_{i=1}^{N_{C}}({\bar{G}}_{iV}-{\bar{G}}_{iL})dN_{iV}$$

when a system reaches equilibrium, the Helmholtz free energy reaches its minimum value, which is represented as dA=0. This can be transformed into the expression for fugacity:

(8) $$f_{i}^{L}=f_{i}^{V}$$

(9) $$x_{i}\varphi_{i}\left(x_{i},P^{L},T\right)P^{L}=y_{i}\varphi_{i}\left(y_{i},P^{V},T\right)P^{V}$$

where $\varphi_{i}$ represents the fugacity coefficient of component *i*, generally expressed as

(10) $$\ln\varphi_{i}=\frac{1}{RT}\int_{V}^{\infty }\left[{\left(\frac{\partial P}{\partial n_{i}}\right)}_{T,V,n_{j}\left(i\neq j\right)}-\frac{RT}{V}\right]dV-\ln Z$$

where $\varphi_{i}$ is the fugacity coefficient of the corresponding component, *R* is the gas volume constant, *Z* is the compressibility factor, *T* is the system temperature, *P* is the system pressure, and V is the volume occupied by the system.

Expanding Equation (10) can yield the calculation expression for fugacity coefficient:

(11) $$\varphi i=e^{\left(-\ln\left(Z-B\right)-\frac{A}{2\sqrt{2}B}\left(\frac{2\sum_{j=1}^{n_{c}}A_{ij}c_{ij}}{A}-\frac{B_{i}}{B}\right)\ln\left(\frac{Z+\left(1-\sqrt{2}\right)B}{Z+\left(1+\sqrt{2}\right)B}\right)+\frac{B_{i}}{B}\left(Z-1\right)\right)}$$

where

$$A_{ij}=\left(1-k_{ij}\right){\left(A_{i}A_{j}\right)}^{0.5},A_{i}=0.45724{\left(1+m\left(1-{T_{r}}^{0.5}\right)\right)}^{2}\frac{P_{ri}^{LV}}{T_{ri}^{2}},B_{i}=0.07778\frac{P_{ri}^{LV}}{T_{ri}}$$

where *k*_*ij*_ is the binary interaction parameter, *T*_*r*_ is the reduced temperature, and *P*_*r*_ is the reduced pressure. For simplicity of calculation, it is assumed that the total molar quantity N = 1 mol for each calculation. Therefore, in equilibrium calculations, material balance must also be satisfied:

(12) $$n^{L}x_{i}+n^{V}y_{i}=N_{i}$$

$x_{i}$ and $y_{i}$ are the mole fractions of the liquid phase and gas phase, respectively, and $n^{L}$ and $n^{V}$ correspond to the molar quantities of the liquid phase and gas phase. When the system reaches equilibrium, the distribution of each component in the two phases should reach equilibrium and satisfy material conservation. Therefore, introducing equilibrium constants, satisfying

(13) $$x_{i}=\frac{c_{i}}{1+n^{V}\left(K_{i}-1\right)}$$

(14) $$y_{i}=\frac{c_{i}K_{i}}{1+n^{V}\left(K_{i}-1\right)}=K_{i}x_{i}$$

Organizing, we get

(15) $$\sum_{i=1}^{n_{c}}\left(y_{i}-x_{i}\right)=0$$

(16) $$f\left(n^{V}\right)=\sum_{i=1}^{n_{c}}\frac{c_{i}\left(K_{i}-1\right)}{1+n^{V}\left(K_{i}-1\right)}=0$$

This set of equations contains 2Nc+2 unknowns (Nc liquid phase mole fractions $x_{i}$, Nc gas phase mole fractions $y_{i}$, and the phase ratio of the two phases: $n^{L}$ and $n^{V}$). Correspondingly, solving the set of equations involves 2Nc+2 equations for closure, and all the equations are solved nonlinearly.

In addition, the model considers restricted fluid calculations, so capillary forces and critical parameter corrections need to be added in the calculation process.

In the critical point transition, the critical point calculation method proposed by Zarragoicoechea et al. [41] is used in this study, and the critical point of the mixture is calculated through a mixing rule.

(17) $$\Delta T_{c}=0.9409\frac{\sigma_{LJ}}{R_{p}}-0.2415{\left(\frac{\sigma_{LJ}}{R_{p}}\right)}^{2}$$

(18) $$\Delta P_{c}=0.9409\frac{\sigma_{LJ}}{R_{p}}-0.2415{\left(\frac{\sigma_{LJ}}{R_{p}}\right)}^{2}$$

where σ and $R_{p}$ are Lennard–Jones size parameters and pore radius.

The model corrects the capillary force by calculating the Young–Laplace equation and then derives the changes in oil and gas phase pressure and fugacity coefficients. For adsorption phenomena, this paper refers to a description method proposed by Song et al. in 2020 [42], which calculates the adsorption layer thickness by establishing a columnar model using isothermal adsorption. In the calculation process, $R_{p}-2\gamma$ is taken as the actual pore throat radius. The thickness of the adsorption layer is given by

(19) $$\gamma =\frac{m}{\ln\left(\frac{R_{p}}{\sigma_{LJ}}\right)}+n\frac{\sigma_{LJ}}{R_{p}}$$

where *m* is the correction factor in the equation of state.

Therefore, the capillary force can be obtained as

(20) $$P_{cap}=\frac{2\sigma \cos\theta }{R_{p}-2\gamma }$$

Finally, by formulating appropriate iterative variables to solve the above nonlinear equations, all calculation steps are completed, and a convergence check is performed on the results.

## 3. Model Validation

To verify the accuracy of the model, the composition data of the B79, B131, and B18 block formations were taken into account, as shown in Table 2. The selected blocks, B79, B131, and B18, represent reservoirs with different lithological and pore structure characteristics. The B79 block primarily consists of tight sandstone with moderate porosity and permeability, while the B131 and B18 blocks contain a mix of carbonate and sandstone formations with varying degrees of heterogeneity. These blocks were chosen due to their representative geological conditions for studying gas injection and phase behavior, covering a range of pore structures, including nano- and micro-scale pores, which are typical in unconventional reservoirs. The optimal grouping method was determined through injection expansion experiments. On this basis, a contact experiment and multiple contact experiments were conducted to investigate the calculation performance of the model in terms of component, pressure, and volume parameters, and to assess the model’s accuracy in predicting phase behavior under varying reservoir conditions. The main gases selected include CO_2_ and natural gas, while the reservoir fluids consist of the formation oils from the aforementioned three blocks.

### 3.1. Grouping of Components

In model calculations, the required initial data include temperature, pressure, components, and basic phase parameters of components such as critical properties and binary interaction coefficients. The component parameters can be obtained through oil and gas chromatography data, but if all components are included in the calculation, it may lead to non-convergence or excessive computational burden. Therefore, the best grouping method is selected by comparing the consistency of the results obtained from different component splitting calculations with the injection experiment, which provides a basis for subsequent calculations. The specific experimental steps are as follows.

The full window high-pressure PVT analyzer is cleaned under reservoir temperature conditions and then evacuated. A certain amount of crude oil sample is transferred to the PVT analyzer to maintain a single-phase state at reservoir temperature for 8 h. First, the sample volume is tested under reservoir pressure, and then a certain amount of CO_2_ is injected into the reservoir crude oil at this pressure to increase the system pressure until all the CO_2_ is dissolved, at which point the system becomes single-phase. Parameters such as saturation pressure and volume expansion coefficient of the CO_2_-reservoir crude oil system are tested. Finally, the CO_2_-reservoir crude oil mixture sample in the PVT analyzer is transferred to the high-temperature and high-pressure electromagnetic viscometer to test the viscosity of the system in a single phase. This completes the first gas expansion experiment. The gas injection amount in the next level of the experiment is higher than that in the previous level (10% higher). This process is repeated, and a total of seven gas expansion experiments are conducted at reservoir temperature.

**Figure 1:**
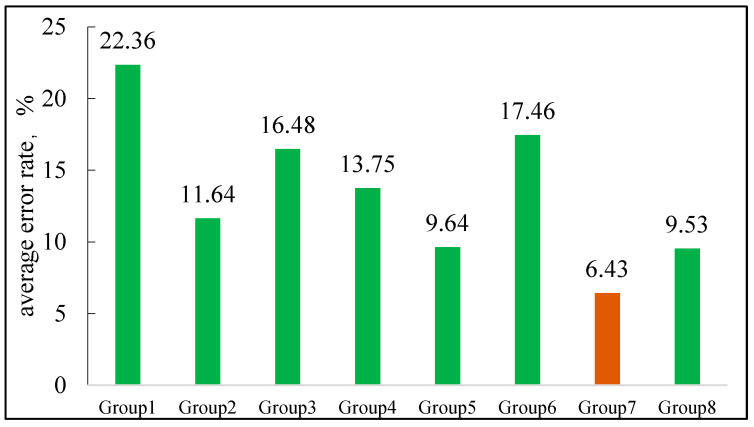
The average error rate of model calculation and experimental results under different grouping methods.

**Figure 2:**
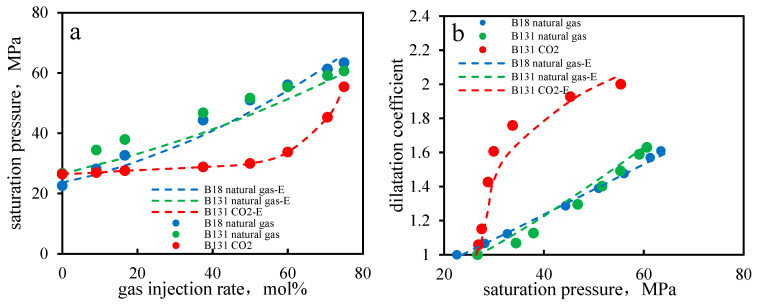
Comparison of phase envelope predictions: Helmholtz-based model provides improved accuracy under confinement. (**a**) Saturation pressure (**b**) expansion coefficient.

A large amount of data on different component hydrocarbon systems at different pressures were obtained through experiments, and a total of eight grouping methods were used to group the components (as shown in Table 3). The best grouping method was selected as the seventh group in Figure 1. Based on this optimal result, calculations were performed for the B131 and B18 reservoir oils and compared with experimental results, as shown in Figure 2 (the calculations were conducted using the phase equilibrium equations (Equations (11)–(14)) presented in Section 2).

### 3.2. Experimental Verification

#### 3.2.1. Experimental Procedure for Single-Contact Mass Transfer Experiment

**Figure 3:**
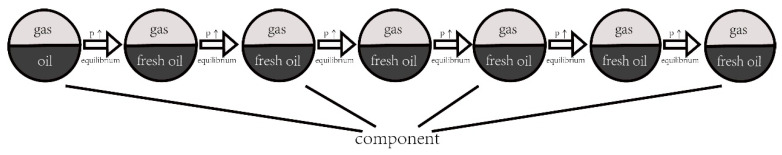
Phase envelope shrinkage of B131 formation oil at different pore sizes.

The specific experimental procedure is shown in Figure 3. By injecting reservoir oil and a sufficient amount of injection gas into the high-temperature and high-pressure PVT apparatus, the pressure is gradually increased above the reservoir oil saturation pressure. After reaching equilibrium, the gas and oil phase components are analyzed by gas chromatography to investigate the changes in the composition of the two phases. The experimental temperature is 90 °C, and the B131 reservoir oil is selected as the liquid phase component, while CO_2_ is used as the injection gas. The pressure is gradually increased (from 26 MPa to 38 MPa), and the equilibrium oil and gas phase compositions are separately analyzed.

**Figure 4:**
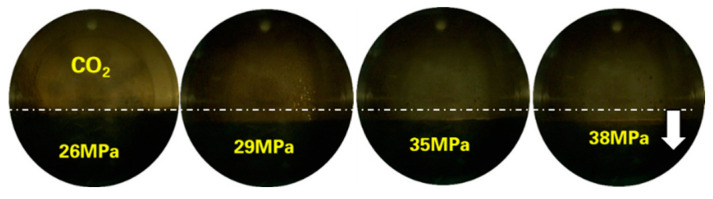
Morphology of CO_2_ and B131 formation oil in PVT as pressure increases.

**Figure 5:**
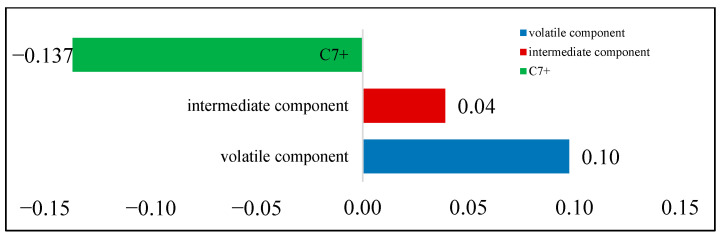
Gas phase component gradient with pressure.

Taking the B131 experiment as an example, experimental photos of the interaction between CO_2_ and reservoir oil were captured. The morphology of CO_2_ in the PVT experiment is shown in Figure 4. The gradient of gas phase components with pressure change is shown in Figure 5, with volatile components at 0.0975 mol%/MPa, intermediate components at +0.039 mol%/MPa, and C_7+_ components at −0.137 mol%/MPa. The gas phase volume gradually increases, the color deepens, and the phase boundary between the two phases becomes blurry. This indicates that the extraction effect is stronger than the dissolution effect in the process of single-contact mass transfer of CO_2_, which is related to the content of intermediate hydrocarbon components such as C_2_–C_6_ in the reservoir oil. Combining the component data in Table 2, it is not difficult to find that the B131 reservoir oil contains a large number of light components and intermediate hydrocarbons.

##### Calculation Results of Component Parameters

**Figure 6:**
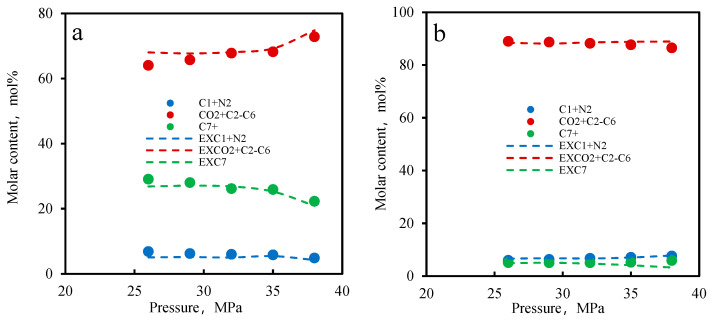
B131 formation oil-CO₂ system: liquid and gas phase composition changes with pressure. (**a**) liquid phase (**b**) gas phase.

In order to verify the accuracy of the model’s component calculations, the oil and gas phase components in the experiment were calculated at the corresponding experimental pressure. The calculation was based on a gas–liquid substance ratio of 2:1 to 3:1 to ensure that the volume ratio of the oil and gas phases at the initial pressure was 1:1. The calculation results are shown in Figure 6 (the phase equilibrium calculations in this figure were conducted using the state equations (Equations (13)–(14)) and equilibrium equations (Equations (16)–(21)) from Section 2). The calculation results and experimental results show a good agreement, with a component error control within 5%, indicating that the model accurately calculates the oil and gas components. This error margin arises from multiple factors, including measurement precision in the PVT analysis, minor variations in fluid composition, and the simplifications inherent in the thermodynamic model, such as the grouping of hydrocarbon components and the use of binary interaction coefficients. Furthermore, similar studies on phase equilibrium calculations typically report deviations ranging from 3% to 6%, suggesting that our results are within a reasonable range for engineering applications. Although minor discrepancies exist, they do not significantly impact the overall phase behavior trends, which are the primary focus of this study in assessing CO₂ injection efficiency. This also demonstrates that the current grouping division of oil and gas phase components has a positive effect on the calculations, which helps improve the accuracy of the calculations.

##### Calculation Results of Volume Parameters

**Figure 7:**
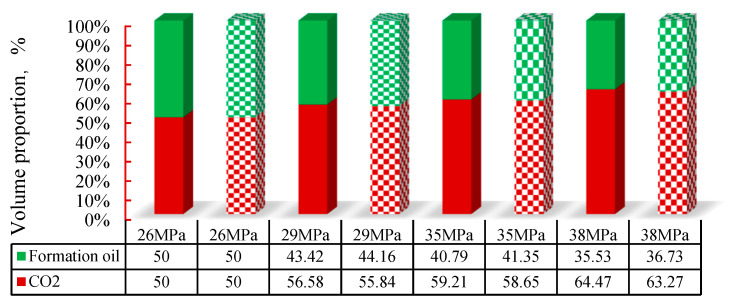
Bubble point pressure variation with decreasing pore size.

By calculating the area of Figure 4, the volume ratio of the oil and gas phases of the M131 formation oil-CO_2_ was estimated at different pressures. The comparison between the estimated results and the calculated results is shown in Figure 7 (the volume calculations were performed based on the state equations (Equations (13)–(14)) and volumetric balance equations (Equations (16)–(23)) from Section 2). The solid-colored region represents the area calculated from the photographs taken in the experiment, while the square-filled region represents the calculated volume ratio of the oil and gas phases. The error in volume calculation for the CO_2_-multiple hydrocarbon system is less than 4%, indicating strong consistency between the model predictions and experimental observations. This deviation is primarily due to uncertainties in experimental volume measurements, as well as the assumptions made in the equation of state modifications. Given that previous studies report volume prediction errors of approximately 3–6% in similar thermodynamic calculations, our model’s accuracy remains within an acceptable range. More importantly, the overall trends in phase behavior remain consistent, ensuring that the model can be effectively used for analyzing CO₂ injection in unconventional reservoirs This indicates that the computational model constructed in the second part effectively captures the influence of pressure changes on the phase transition of fluids at conventional scales.

#### 3.2.2. Multistage Contact Experiments and Component Parameter Calculation Results

The mixing of CO_2_ and formation oil is divided into forward multi-stage contact mixing (evaporative mixing) and backward multi-stage contact mixing (condensation mixing). In this experiment, forward and backward multi-stage contact mixing experiments were conducted to simulate the process of gas enrichment or oil depletion during injection. Based on the accurate validation of a single contact experiment, the accuracy of the model calculations under fluid composition changes was further validated through multi-stage contact experiments.

**Figure 8:**
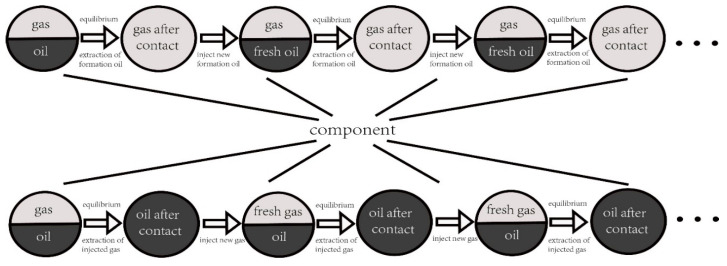
Saturation pressure reduction in confined nanopores.

The specific method is illustrated in Figure 8. In the forward contact experiment, fresh formation oil and injected gas were mixed, and then the resulting liquid phase was removed. This process was repeated to investigate the changes in gas phase composition. On the contrary, in the backward contact experiment, fresh injected gas was brought into contact with the formation oil, and the resulting gas phase was removed. Fresh injected gas was then introduced, and this process was repeated to investigate the changes in the oil phase. In this experiment, B131 block formation oil was selected as the liquid phase, and natural gas (95% C_1_ + 5% C_2_) was chosen as the gas phase. The experimental pressure was 33.1 MPa, and a total of four forward contact experiments and backward contact experiments were conducted.

**Figure 9:**
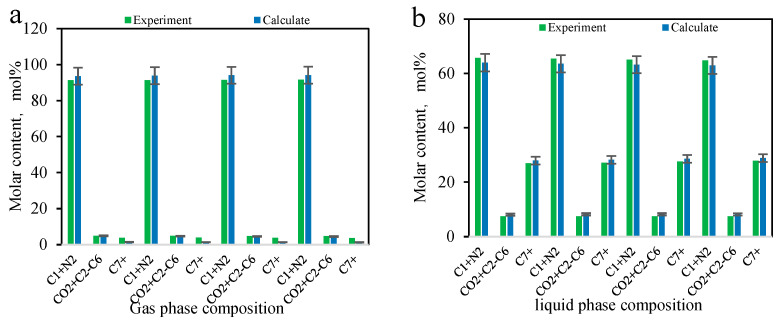
Comparison of composition changes in multi-stage contact experiments and model calculation results (**a**) liquid phase (**b**) gas phase. (The calculations were performed using the forward method.)

**Figure 10:**
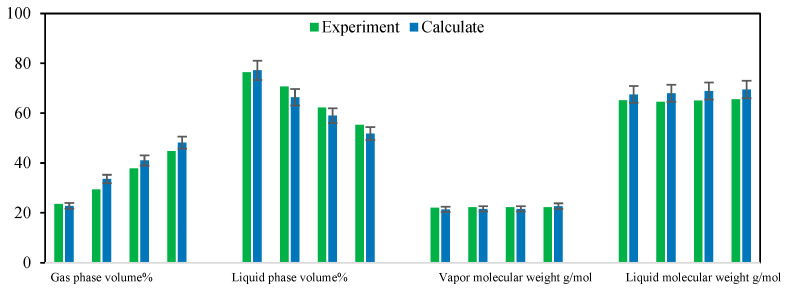
Comparison of gas/liquid phase volume and molecular weight changes in multi-stage contact experiments with model calculation composition changes. (The calculations in this figure were performed using the forward method.).

**Figure 11:**
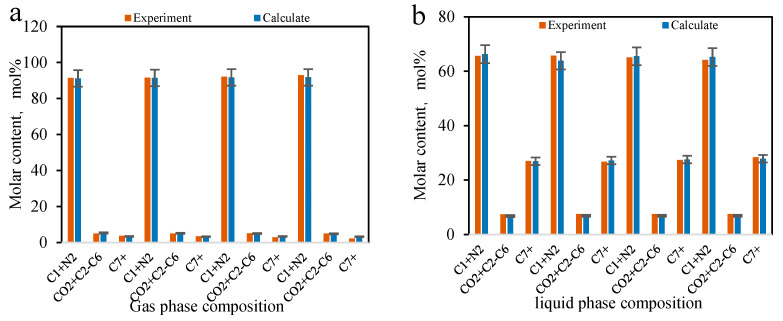
Comparison of liquid phase composition changes in multi-stage contact experiments and model calculation results (**a**) liquid phase (**b**) gas phase. (The calculations in this figure were performed using the backward method.)

**Figure 12:**
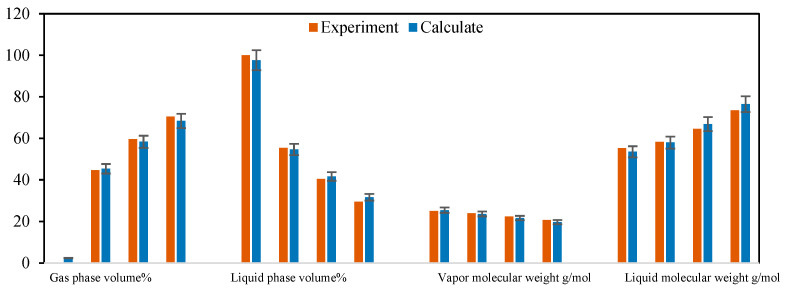
Comparison of gas/liquid phase volume and molecular weight changes in multi-stage contact experiments with model calculation composition changes. (The calculations in this figure were performed using the backward method.)

The results of the multi-stage contact experiments and calculations for the B131 formation oil–gas are shown in Figure 9 and Figure 10 (the calculations used in this figure are based on the phase equilibrium equations (Equations (11)–(14)) and equilibrium expressions (Equations (16)–(21)) from Section 2) for forward multi-stage contact and in Figure 11 and Figure 12 for backward multi-stage contact. The errors in the gas and liquid phase component compositions for multiple contacts were controlled within 3%. The calculated gas and liquid phase volumes and molecular weights were compared with the results from multiple contact experiments, with errors controlled within 10%. These results demonstrate that the model exhibits good stability and accuracy in calculating the fugacity coefficient, volume, and viscosity for CO_2_-hydrocarbon systems. The model is capable of simulating the mass transfer process of component exchange in actual oil and gas multiple contact scenarios.

### 3.3. Literature Comparison and Verification

**Figure 13:**
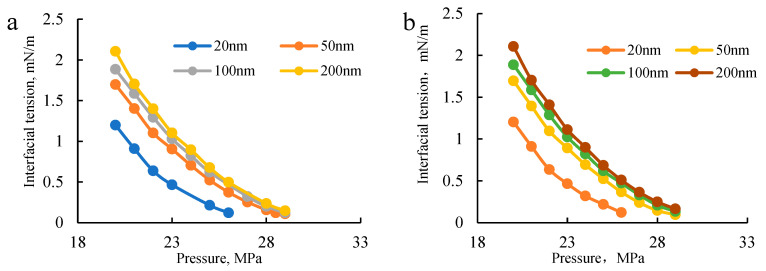
(**a**) Gas–liquid interfacial tension vs. scale variation [42]. (**b**) Model repeated calculation of gas–liquid interfacial tension versus scale change and the verification effect of Wu et al.

At the conventional scale, the model has been validated through gas injection expansion, single-stage contact mass transfer, and multi-stage contact experiments. Under restricted conditions, this study also compared the model predictions with recent research findings. Han et al. [43] investigated the phase behavior of oil-CO_2_-water systems under water-alternating-gas (CO_2_-WAG) injection and found that the saturation pressure of the oil-CO_2_-water system was lower than that of the oil-CO_2_ system due to partial CO_2_ dissolution in water, which led to a shift in the phase envelope. The model predictions in this study exhibit a similar trend, further confirming the accuracy of the proposed model. Additionally, this study selected data from the article published by Wu et al. in 2020 [42] for repeated calculation and verification, as shown in Figure 13. The calculated gas–liquid interfacial tension at 20 nm, 50 nm, 100 nm, and 200 nm was found to be consistent with the results reported in the literature, indicating that the thermodynamic calculation model developed in this study can meet the computational requirements for restricted fluids.

Through experimental validation and a literature comparison, the model has been thoroughly verified. By grouping the fluid components in the target reservoir, a foundation has been laid for subsequent phase equilibrium calculations at multiple scales. Validation has been carried out through single-stage contact experiments and multi-stage contact experiments to verify important parameters such as component composition, volume, and pressure. At the conventional scale, the model meets the computational requirements. However, it is important to note that experimental measurements have inherent uncertainties, and empirical parameters, such as binary interaction coefficients and isoenthalpic compressibility, for the pseudo-component fluid also introduce certain errors. These errors are in line with the characteristics of thermodynamic models, and it can be considered that the model has a certain level of accuracy. By comparing with the literature, the calculation of restricted fluids at the nanoscale has been validated. The model generally agrees with the literature and satisfies the computational requirements.

## 4. Results and Discussion

In order to investigate the phase behavior of oil and gas at multiple scales, this section presents the calculations and diagrams of phase diagrams for four different fluids, namely B131, B18, B79, and B18-CO_2_ (1:1), at sizes of 10 nm, 50 nm, 100 nm, 150 nm, and 200 nm, as well as at bulk phase. Taking B79 reservoir oil as an example, three aspects, including density expansion, differential separation, and multi-stage contact simulation, were explored to comprehensively analyze the possible phase behavior of reservoir fluids in restricted spaces.

### 4.1. Phase Diagram Analysis

**Figure 14:**
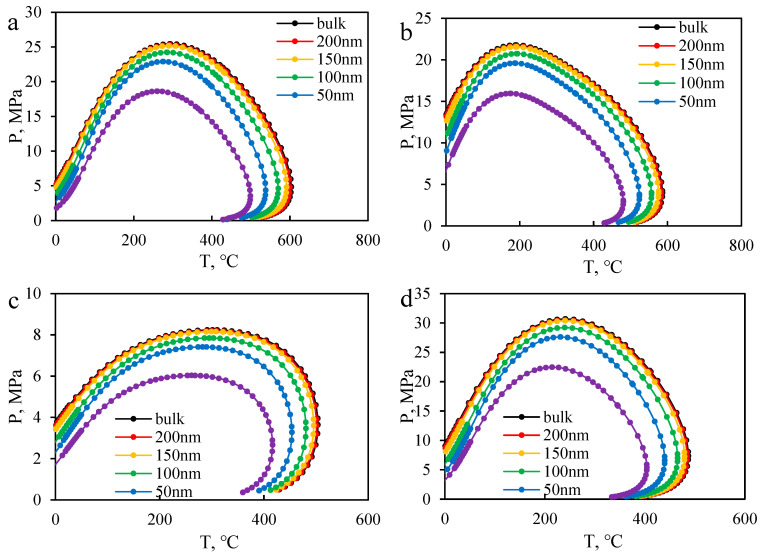
The PT phase envelope of formation oil changes at different scales (**a**) B131 (**b**) B18 (**c**) B79 (**d**) B18 -CO_2_.

**Figure 15:**
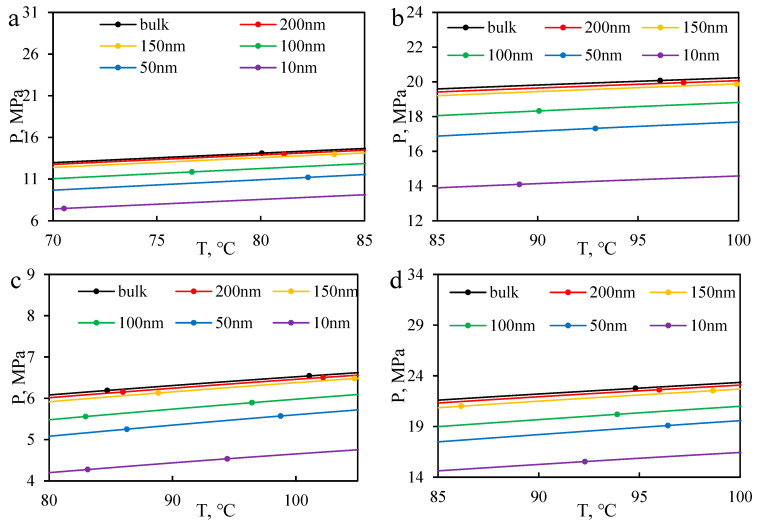
Enlarged results of PT phase envelopes at different scales of fluid near the formation temperature (**a**) B131 (**b**) B18 (**c**) B79 (**d**) B18-CO_2_.

By controlling the fugacity, the bubble and dew points at different pressures were determined, and a large amount of bubble and dew point data were obtained through iterative calculations. The fluid phase diagrams for B131, B18, B79, and B18-CO_2_ (1:1) at different scales are shown in Figure 14 and Figure 15. When the size is larger than 150 nm, the phase envelope diagram remains essentially unchanged. However, when the size is smaller than 150 nm, the envelope lines contract towards the central region. When the scale decreases to 100 nm, the fluid phase envelope diagram exhibits significant contraction, and when the scale decreases to 10 nm, the phase envelope diagram contracts by an average of around 28%, as shown in Figure 14. This contraction indicates a significant shift in bubble and dew point pressures, which suggests that phase equilibrium conditions in nanopores deviate substantially from those in bulk conditions. Notably, Figure 15 further highlights that this contraction effect is more pronounced for higher gas content systems (e.g., B18-CO₂), where gas–liquid interactions dominate phase behavior. Recent studies have also explored the phase behavior of CO_2_ in nanoporous media. Wan et al. [44] found that CO_2_ solubility decreases in smaller pores, and the phase envelope contraction effect becomes more pronounced. This aligns with our findings, as shown in Figure 14, where the phase envelope reduction reaches 28% in pores below 10 nm. These results indicate that traditional bulk-phase models may not accurately predict phase behavior in nano-scale reservoirs, necessitating scale-dependent thermodynamic models for improved predictions. Their study showed that the phase envelope shrinkage reached 20–30% for pores smaller than 10 nm, which aligns well with the simulation results of this study, further validating the model’s applicability. These findings have important implications for reservoir management and CO_2_ injection strategies. The observed phase envelope contraction at nanoscale indicates that phase separation and miscibility conditions in tight reservoirs may significantly differ from those predicted by conventional bulk-phase models. This suggests that for CO_2_-EOR processes in nano-scale reservoirs, injection pressures may need to be adjusted to ensure optimal miscibility and maximize oil displacement efficiency. Furthermore, the reduced bubble point pressure in confined systems implies that gas liberation and exsolution mechanisms will be delayed, which could influence production strategies in ultra-low permeability reservoirs. These insights can help refine reservoir simulation models and improve the design of CO_2_ injection schemes in unconventional formations. Furthermore, compared to B18 reservoir oil and B18 reservoir oil mixed with CO_2_ at a ratio of 1:1, the bubble point pressure increases relatively with the increase in gas phase. In terms of component composition, as the proportion of gas phase increases (B79 < B18 < B131), the influence of scale decreases, and the magnitude of the decrease in bubble point pressure at 10 nm increases (23%~31%), indicating a more significant impact of restricted fluids on the gas phase. Compared to conventional EOS-based models that introduce empirical capillary pressure corrections to account for nanoscale effects, the Helmholtz free energy-based model naturally incorporates these confinement effects into the thermodynamic potential. This allows for a more accurate prediction of bubble point pressure shifts, density variations, and interfacial tension changes in nanoporous media. Unlike conventional approaches, which require separate parameter tuning to fit experimental data, this model inherently captures the thermodynamic constraints imposed by nanoscale confinement, making it more reliable for confined phase behavior modeling. The simulation results demonstrate that the predicted phase envelope shrinkage aligns well with experimental trends, further validating the robustness of this approach.

### 4.2. Density Expansion Simulation

**Figure 16:**
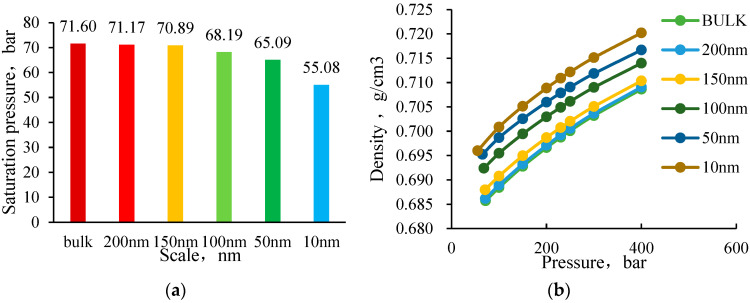
(**a**) Variation law of fluid saturation pressure at different scales in the equilibrium mass expansion process. (**b**) Variation law of fluid density at different scales for equilibrium mass expansion.

Reservoirs suitable for CO_2_-enhanced oil recovery (EOR) often have poor permeability and slow pressure transmission. As a result, the reservoir fluids in some pore throats undergo a pressure drop process. Before effective flow is established, the reservoir fluids are confined in closed spaces, and the phase behavior of the fluids at different scales varies with the pressure drop process. Therefore, it is necessary to conduct a density expansion simulation for this purpose. In this section, the B79-CO_2_ system is taken as an example to simulate the pressure drop and density expansion and to explore the phase behavior of the fluid at different scales. The simulation results are shown in Figure 16. As the scale decreases, the saturation pressure of the reservoir fluid gradually decreases, especially when it is below 100 nm, the deviation in saturation pressure can reach around 5% to 23%, as shown in Figure 16. This suggests that confined fluids in nanopores exhibit a delayed gas liberation effect, which has direct implications for CO_2_ injection strategies in tight reservoirs. Figure 16a further illustrates how saturation pressure changes across different scales, showing a more significant deviation below 50 nm. Additionally, Figure 16b presents the variation in fluid density, revealing that confined fluids exhibit an increased density of approximately 2% compared to bulk conditions. These results emphasize the importance of considering nanoscale effects when designing CO_2_ injection schemes, as failure to account for these shifts could lead to suboptimal reservoir performance predictions. This indicates that the fluids in smaller pores have more difficulty degassing. Compared to the BULK state, the fluid density increases by an average of 2%, and the volume decreases by an average of 2% for sizes below 10 nm. Density variation is a crucial phenomenon in nanopores. Pan et al. [45] demonstrated that, in CO₂-hydrocarbon systems, the confinement effect at the nanoscale results in a 1.5–2.8% increase in gas–liquid density compared to bulk conditions. The experimental results in this study indicate a density expansion ratio of approximately 2% within the 10 nm pore size range, further verifying the universality of this trend. In terms of lateral comparison, as the scale decreases below 50 nm, the compressibility factor and compressibility of the fluid also undergo significant changes. In restricted spaces, the ability of the fluid to degas and expand is weakened. This means that the fluid in the pore may exhibit gas–liquid two-phase behavior before passing through the throat, leading to the Jamin effect. Additionally, when passing through the throat, the gas phase may dissolve back into the liquid phase, thereby weakening or even eliminating the J effect caused by partial degassing.

### 4.3. Differential Separation Simulation

**Figure 17:**
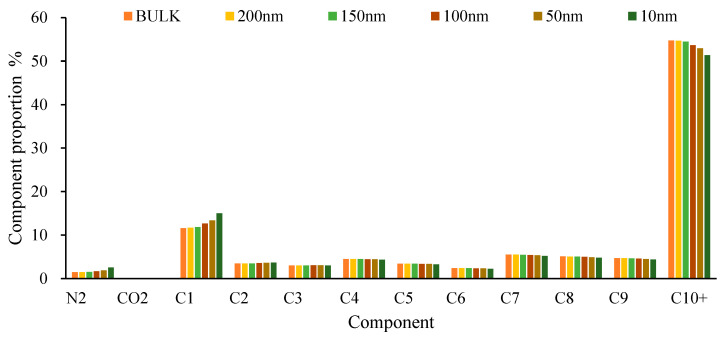
Scale-dependent variation of liquid phase composition at 50 bar increased retention of heavier hydrocarbons in smaller pores.

**Figure 18:**
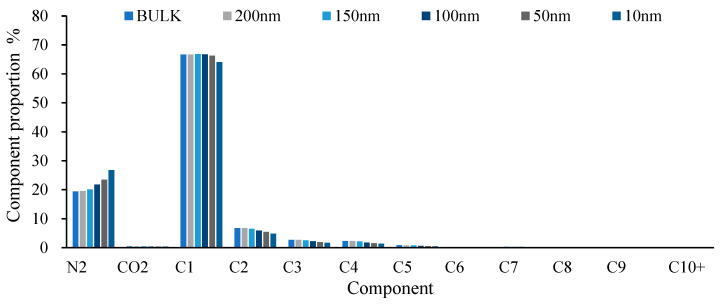
Scale-dependent variation of gas phase composition at 50 bar enhanced CO_2_ retention and depletion of lighter hydrocarbons in smaller pores.

As the pressure depletion production progresses and gas–liquid separation occurs, the fluid composition within the near-well matrix undergoes changes, as shown in Figure 17 and Figure 18. At 50 bar, the proportion of C_1_ components in the liquid phase increases significantly at 10 nm and 50 nm, while the proportions of C_1_–C_4_ components in the gas phase decrease and the proportion of N_2_ increases. This indicates that during the degassing process, N_2_, which is more difficult to dissolve, is preferentially removed, while C_1_, as the main dissolved gas component, decreases in proportion, indicating that a considerable amount of C_1_ remains in the liquid phase. This also explains why the density of the liquid phase decreases as the scale decreases.

### 4.4. Multi-Stage Contact Simulation

**Figure 19:**
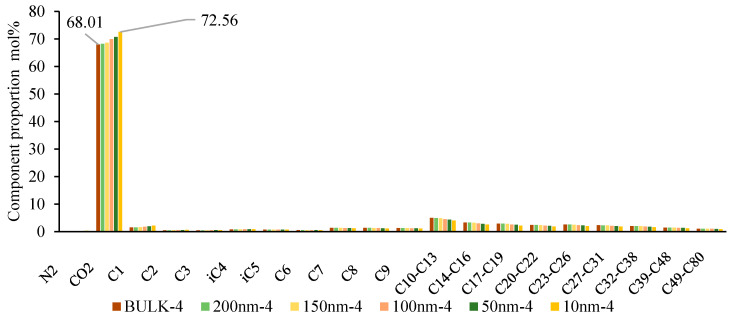
Variation of liquid phase components at different scales in the fourth stage of backward multi-stage contact process.

**Figure 20:**
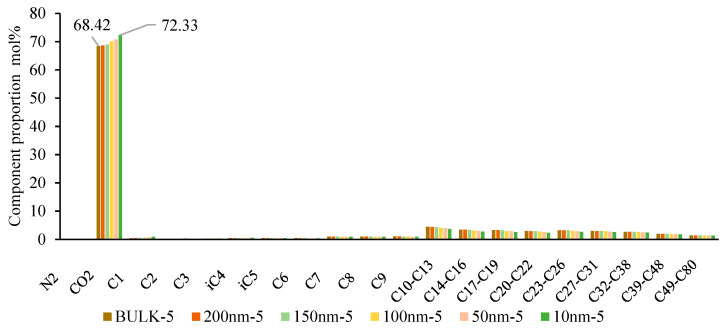
Variation of liquid phase components at different scales in the fifth stage of backward multi-stage contact process.

**Figure 21:**
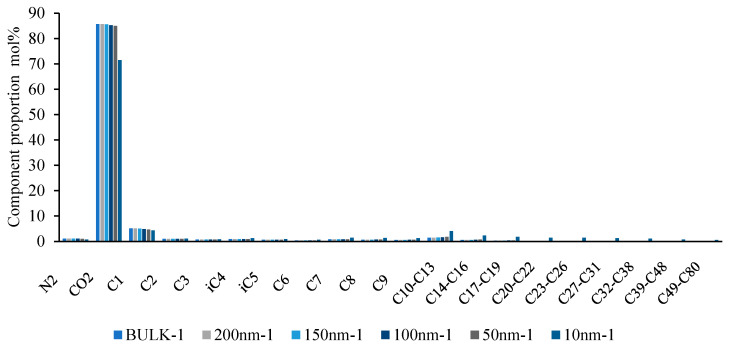
Variation of gas phase components at different scales in the first stage forword multi-stage contact process.

The basis of multi-stage contact mixing is the exchange and mass transfer of components between the two-phase fluids. Therefore, understanding the variations in fluid composition at different scales is crucial in describing the phase behavior changes during multi-stage contact. As shown in Figure 19, Figure 20 and Figure 21, in the backward multi-stage contact simulations for the fourth and fifth stages, as the scale decreases, the CO_2_ content in the liquid phase gradually increases (from 69.01% to 72.56% and from 68.42% to 72.33%), while the content of C_2_-C_5_ components also increases, and the content of C_7+_ decreases. This indicates that as the scale decreases, the ability of CO_2_ to dissolve into the reservoir oil is enhanced, and the reservoir oil retains gas phase components more effectively. The mass transfer between the gas and liquid phases becomes more significant, and the properties of the liquid phase approach those of the CO_2_ phase. Consequently, phase mixing becomes easier, which is consistent with numerous recent research findings. In the forward multi-stage contact simulations, after one contact at 100nm and 50nm, the oil and gas phases form a single-phase state after the second contact. However, at 10nm, a single-phase state is achieved after the first contact. This indicates that phase mixing formed by gas extraction and stripping is easier compared to the phase mixing formed by CO_2_ dissolution.

## 5. Conclusions

(1) Based on the traditional thermodynamic theory, the thermodynamic fluid phase equilibrium calculation model was established by introducing the Helmholtz free energy and considering capillary force, critical point transition, and adsorption. This model provides a new approach for calculating phase parameters of oil and gas at multiple scales. It clarifies the limits (around 200 nm) and extent (2.5 MPa pressure drop for oil–gas mixing at 50 nm) of changes in the phase properties of oil and gas under spatial variations (PVT cylinder and porous media). The theoretical analysis also reveals the phase characteristics changes in the CO_2_ displacement front, oil–gas mixing zone, diffusion zone, and pressure drop zone within the porous media. Unlike conventional Gibbs free energy models, which require empirical corrections for nanoscale effects, the Helmholtz free energy model inherently integrates capillary pressure and adsorption effects, leading to more accurate phase behavior predictions and improved computational stability.
(2) Taking B131, B18, and B79 reservoir oil components as examples and combining them with gas injection expansion experiments for component splitting, the model was validated by comparing the results of single-stage contact experiments, multiple-stage contact experiments, and previous research findings. The results confirm that the model provides accurate calculations for both bulk and nanoscale systems.
(3) Using a B79-CO_2_ displacement block in a specific oilfield as an example, the phase behavior of the fluid during the CO_2_ injection process was analyzed through phase diagram analysis, density expansion simulation, differential separation simulation, and multi-stage contact simulation. The results clarify the phase characteristics of the fluid under different scales. As the scale decreases (from 200 nm to 10 nm), the fluid experiences enhanced confinement effects, making gasification more difficult in the liquid phase and resulting in an increase in overall density (1.7% increase at 10 nm). The mass transfer and phase mixing abilities between CO_2_ and reservoir oil increase with decreasing scale. Under the condition of sufficient contact between oil and gas, the reduction in scale has a positive effect on improving the oil-washing efficiency of CO_2_. These findings indicate that scale-dependent phase behavior should be considered in CO_2_-EOR designs. For ultra-tight formations, adjusting CO_2_ injection rates and optimizing contact time based on the scale of nanopores can enhance oil recovery efficiency. Field data integration with the model can provide actionable insights for designing site-specific injection strategies.
(4) Application to CO_2_-EOR optimization: The findings of this study provide practical guidelines for optimizing CO_2_ injection strategies in unconventional reservoirs. The observed phase behavior shifts suggest that higher injection pressures (e.g., above 15 MPa for nanoporous reservoirs) are critical to maintaining miscibility and preventing early gas liberation. Additionally, the enhanced CO_2_ solubility at nanoscale indicates that Continuous injection is recommended over cyclic methods like WAG for nanoporous formations due to enhanced CO_2_ solubility. For field implementation, the proposed model can be embedded in reservoir simulators to refine injection schedules and predict phase transitions under confined conditions. For example, simulations can identify optimal injection pressure ranges tailored to specific pore size distributions. This approach aids in designing injection schedules that optimize miscibility windows and minimize CO_2_ loss. Moreover, integrating this model with real-time monitoring data can enhance dynamic adjustment strategies for CO_2_ flooding efficiency in heterogeneous formations. This model can assist in fine-tuning injection pressures and predicting phase stability across multiple scales, contributing to improved oil recovery efficiency in complex porous media.

## Figures and Tables

**Table 2:** Formation oil components of BLOCK B131, B18, B79.

Component	B79	B131	B18
CO_2_	0.153	0.252	0.208
N_2_	2.818	3.708	3.06
C_1_	16.193	53.522	44.168
C_2_	3.938	3.112	2.568
C_3_	3.224	0.716	0.591
iC_4_	1.675	0.283	0.234
nC_4_	2.978	0.219	0.181
iC_5_	0.904	0.124	0.102
nC_5_	2.594	1.358	1.7
C_6_	2.431	2.066	2.625
C_7_	3.835	3.503	4.507
C_8_	5.131	4.203	5.404
C_9_	4.225	3.754	4.83
C_10_	3.897	3.825	4.921
C_11_	3.36	2.119	2.727
C_12_	3.256	2.979	3.833
C_13_	3.271	1.976	2.543
C_14_	2.697	1.784	2.295
C_15_	2.746	1.242	1.599
C_16_	2.208	1.561	2.008
C_17_	2.249	1.291	1.661
C_18_	1.999	0.804	1.034
C_19_	1.921	0.637	0.819
C_20_	1.764	0.484	0.623
C_21_	1.604	0.483	0.622
C_22_	1.554	0.414	0.533
C_23_	1.431	0.367	0.472
C_24_	1.385	0.303	0.389
C_25_	1.232	0.286	0.368
C_26_	1.153	0.254	0.327
C_27_	1.062	0.253	0.326
C_28_	1.024	0.219	0.282
C_29_	0.936	0.191	0.246
C_30_	0.905	0.135	0.174
C_31_	0.7	0.101	0.129
C_32_	0.716	0.074	0.095
C_33_	0.548	0.064	0.083
C_34_	0.532	0.067	0.086
C_35_	0.476	0.093	0.12
C_36+_	5.275	1.17	1.506

**Table 3:** Split grouping of components in calculation.

Number	Group1	Group2	Group3	Group4	Group5	Group6	Group7	Group8
G1	CO_2_	CO_2_	CO_2_ + C2	CO_2_	CO_2_	CO_2_	CO_2_	CO_2_
G2	N_2_	N_2_ + C_1_	N_2_	N_2_	N_2_	C_1_	C_1_ + N_2_	N_2_ + C_1_
G3	C_1_	C_2_–C_4_	C_1_	C_1_	C_1_	C_2_	C_2_	C_2_
G4	C_2_	C_5_–C_6_	C_3_–C_4_	C_2_–C_4_	C_2_–C_6_	C_3_	C_3_–C_4_	C_3_–C_4_
G5	C_3_	C_7_–C_13_	C_5_–C_6_	C_5_–C_6_	C_7_–C_13_	C_4_	C_5_	C_5_–C_6_
G6	C_4_	C_14_–C_19_	C_7_–C_13_	C_7_–C_19_	C_14_–C_19_	C_5_	C_6_	C_7_–C_13_
G7	C_5_	C_20_–C_32_	C_14_–C_19_	C_20_–C_25_	C_20_–C_25_	C_6_	C_7_–C_13_	C_14_–C_19_
G8	C_6_	C_33_+	C_20_–C_32_	C_25_–C_32_	C_25_+	C_7_–C_19_	C_14_–C_19_	C_20_+
G9	C_7_+		C_33_+	C_33_+		C_20_+	C_20_–C_32_	
G10							C_33_+	
G11								
G12								

## Data Availability

Not appliable.
